# Sleep and Association With Cardiovascular Risk Among Midwestern US Firefighters

**DOI:** 10.3389/fendo.2021.772848

**Published:** 2021-11-11

**Authors:** Juan Luis Romero Cabrera, Mercedes Sotos-Prieto, Antonio García Ríos, Steven Moffatt, Costas A. Christophi, Pablo Pérez-Martínez, Stefanos N. Kales

**Affiliations:** ^1^ Lipids and Atherosclerosis Unit, Department of Internal Medicine, Maimonides Biomedical Research Institute of Córdoba (IMIBIC), Reina Sofía University Hospital, University of Córdoba, Córdoba, Spain; ^2^ Department of Environmental Health, Harvard T.H. Chan School of Public Health, Boston, MA, United States; ^3^ CIBER Physiopathology of Obesity and Nutrition (CIBEROBN), Carlos III Institute of Health, Madrid, Spain; ^4^ Department of Preventive Medicine and Public Health, School of Medicine, Universidad Autónoma de Madrid; IdiPaz (Instituto de Investigación Sanitaria Hospital Universitario La Paz); and CIBERESP (CIBER of Epidemiology and Public Health), Madrid, Spain; ^5^ National Institute for Public Safety Health, Indianapolis, IN, United States; ^6^ Cyprus International Institute for Environmental and Public Health, Cyprus University of Technology, Limassol, Cyprus; ^7^ Occupational Medicine, Cambridge Health Alliance/Harvard Medical School, Cambridge, MA, United States

**Keywords:** obesity, hypertension, cardiometabolic risk, shift workers, sleep

## Abstract

**Introduction:**

Cardiovascular disease is the leading cause of on-duty fatalities among U.S. firefighters. Research has demonstrated that many modifiable risk factors are contributors to the high prevalence of cardiometabolic risk factors. The current study aimed to assess whether sleep is associated with cardiometabolic risk factors among Indianapolis firefighters. The findings could support improving sleep hygiene in this population.

**Material and Methods:**

This cross-sectional study was conducted from the baseline data of eligible firefighters enrolled in “Feeding America’s Bravest”, a Mediterranean diet lifestyle intervention within the Indiana Fire Departments. Participants’ sleep quality was categorized as “good” (≤ 8 points) or “bad” (>8 points) by a sleep quality questionnaire based on some questions from Pittsburgh Sleep Quality Index. In addition, firefighters’ sleep duration was stratified based on the number of hours slept per night (≤6 as “short sleep” or >6 hours as normal). Linear and logistic regression models were used to examine the association of sleep with cardiometabolic risk factors.

**Results:**

A total of 258 firefighters were included. Bad sleepers had higher weight, greater waist circumference, higher body mass index (BMI), and increased body fat (all p<0.01) compared to good sleepers. Similarly, participants with short sleep duration were heavier (p<0.02), had greater BMI (p<0.02) and increased body fat (p<0.04) compared with participants with normal sleep duration. Both bad and short sleepers had a higher prevalence of hypertension and obesity (p <0.05).

**Conclusions:**

Our study supports that both sleep quality and quantity are associated with cardiometabolic risk among firefighters.

## Introduction

Cardiovascular disease (CVD) remains the leading cause of death worldwide (1). In recent years, the influence of sleep on metabolic and cardiovascular health has become an important, emerging field of research. Current evidence supports that the quality and quantity of sleep are important modifiable lifestyle factors similar to dietary patterns, sedentary behavior, and smoking regarding the development of CVD (2). Poor sleep quality and sleep quantity have been linked with increases in cardiometabolic risk factors, including obesity, hypertension, and diabetes mellitus, and consequently with an increased risk of CVD death (3, 4).

In the U.S., many public safety workers (e.g., police, emergency medical services and firefighting) are shift workers. Cardiovascular disease is the leading cause of on-duty death among US firefighters, and autopsy evidence of coronary heart disease is present among a large majority of on-duty fatalities (5, 6). Additionally, firefighters are a population predisposed to sleep disturbances and sleep disorders (7). These, in turn, are considered major contributors to firefighters’ high prevalence of cardiometabolic risk factors, burnout and other behavioral health issues (8). This lifestyle is in part a consequence of their work schedule, which typically includes 24 hour-shifts and often involves second jobs during the time off from the fire department. Both may challenge firefighters from obtaining adequate sleep. Accordingly, firefighters’ shift work may lead to misalignment and alterations of circadian rhythms, which can be associated with changes in glucose and lipid metabolism, inflammation and autonomic nervous system regulation, thus increasing the risk of atherosclerosis, dyslipidemia and insulin resistance (9). In addition, shift work and the associated sleep disturbances/sleep deprivation are believed to influence the dietary habits of firefighters. Meal times are often unpredictable and inconsistent, which increases the risk of poor dietary choices, including sugar-sweetened beverages and fast-/takeout-foods which are linked to obesity and metabolic syndrome (10–12).

While, there is evidence linking poor quality of sleep on shift workers such as nurses and law enforcement to the risk of chronic diseases (13, 14), few studies have assessed this relationship in firefighters. Firefighters’ shift work is different from that of nurses and police who cover the 24-hours of the day usually in 8-12 hour shifts. Firefighters, on the other hand, typically work 24-hour shifts with highly variable opportunities for sleep and rest during these shifts. Moreover, they often engage in second jobs during their “off-duty” time.

The current cross-sectional study further examined whether the quality and quantity of sleep among Indianapolis firefighters are related to objective cardiometabolic risk factors.

## Material and Methods

### Study Design

“Feeding America’s Bravest” was a prospective, cluster-randomized clinical trial to compare a Mediterranean Diet Nutrition Intervention vs. usual care (control) in career firefighters within the 44 fire stations of the Indianapolis Fire Departments and the 6 fire stations of the Fishers Fire Department, Indiana Fire Departments (15). Over 1000 firefighters were invited to participate, at the beginning of the study 486 participants consented to participate in the trial (enrollment between November 28, 2016 and April 16, 2018). Inclusion criteria for the present study were informed consent, a completed baseline questionnaire and complete anthropometric measures at baseline. Thus, the final sample for the present study was 265 participants.

Feeding America’s Bravest was approved by the Harvard Institutional Review Board (IRB16-0170) and was registered at Clinical Trials (NCT02941757). All firefighters in the current study provided informed consent for participation.

### Baseline Sleep Assessment

According to the Feeding America’s Bravest data collection protocol, a validated food frequency questionnaire (FFQ) and a comprehensive nutritional-lifestyle questionnaire with questions about diet, sleep, tobacco, history of CVD and physical activity behavior were administered. The questions used to assess sleep behavior included in the lifestyle questionnaire were based on some questions from the validated Pittsburgh Sleep Questionnaire Index (PSQI) (16). Based on these questions we constructed a Sleep Quality Index (SQI) (**Supplementary Table 1**) in order to assess sleep quality. The SQI is comprised of 11 items that are scored 0-2 points, with the exception of two questions that are scored 0-3 points. The lowest scores represent the ideal quality for the parameter and the highest, the worst quality. The final SQI score may have a value from 0 to 24, with lower scores indicative of better sleep quality. We then used the SQI scores to categorize participants as good sleepers (≤ 8 points) or bad sleepers (>8 points) based on the median punctuation of SQI.

Furthermore, for sleep duration, three different questions were included in the nutritional-lifestyle questionnaire about sleep per night at home and at the firehouse, and sleep in a typical 24-hour period including naps. Using a definition of short sleep consistent with previous recommendations (17), we dichotomously categorized the participants based on the reported total number of hours slept per night at home and at the firehouse as sleeping 6 or less hours per night, or more than 6 hours per night, and for sleep in a typical 24 hour period, we categorized the participants as sleeping 6 or less hours, and more than 6 hours.

### CVD Risk Factors

Anthropometric measurements (height, waist circumference, body weight, and body fat), as well as blood pressure measurements, were performed by trained medical staff, at the baseline study visit. Height was measured without shoes in the standing position with a standard clinic stadiometer. Waist circumference was assessed by using a tape measure snugly fitted around each participant’s waist at the level of the iliac crest and measuring the circumference after expiration. Body weight was measured with bare feet and in light clothes on a calibrated scale (Tanita). Body fat was estimated using a Bioelectrical Impedance Analyzer (Tanita). Blood pressures were measured using an appropriately sized cuff with each firefighter in the seated position in a resting state.

Hypertension was defined according to the most recent American Hypertension Guideline to having a systolic blood pressure higher than 130 or diastolic blood pressure higher than 80 (18), and we considered obesity to be any body mass index (BMI) greater than or equal to 30 kg/m^2^, as defined by the World Health Organization (WHO) (19) and abdominal obesity was considered as a waist circumference greater than or equal to 102 cm for males and 88 cm for females (20).

### Covariates

A comprehensive nutritional lifestyle questionnaire and a validated FFQ were also collected at baseline of the current study and after 6 months. Information collected included sociodemographic data (age, gender, race, level of education, marital status, and working in a second job). Furthermore, the questionnaire asked specific questions about the frequencies of consumption of different foods and dietary behaviors allowing the computation of the modified Mediterranean Diet Score (mMDS) as previously described (21), along with other questions (including the history of CVD, tobacco smoking, sleep pattern, and physical activity). The mMDS assessed the adherence to a Mediterranean-style dietary pattern and consisted of thirteen items, for each item a scale up to 4 or 5 points was created where the minimum score of 0 represents the choice that least conform Mediterranean diet and the maximum score represents a choice that most conforms to the Mediterranean diet, with a total possible score ranging from 0 (no conformity to a Mediterranean-style diet) to 51 (maximal conformity to a Mediterranean-style diet) as described in previous work in this population. The dietary domains assessed, include the consumption of fast food, fruits, vegetables, legumes, nuts, sweets dessert, fried foods, ocean fish, bread and starches (consumed at home and fire station), the type and frequency of alcoholic beverages, non-alcoholic beverages (consume at home and the fire station) and the type of cooking oil or fat (consumed at home and the fire station) (22) Physical activity was categorized as not participating regularly in programmed recreation, sport, or vigorous physical activity; participating regularly in modest physical activity (such as golf, table tennis, weight-lifting, calisthenics, or gymnastics); or participating regularly in vigorous physical activity (such as running or jogging, swimming, cycling, tennis, basketball).

### Statistical Analysis

Continuous characteristics following normal distributions were presented as means ± standard deviations and compared between groups with the parametric t-test whereas continuous characteristics without normal distributions were shown as median (Q1, Q3) and compared between groups with the use of the Wilcoxon non-parametric test. Categorical variables were presented as counts and percentages and compared between groups using the chi-square test of independence or the Fisher’s exact test, as appropriate.

To examine the association of sleep quality, as measured by the SQI, or sleep quantity with cardiovascular disease risk factors we used linear regression models and present the effect of a one-unit increase in the SQI on these measures after adjusting for potential confounding factors: age, gender, smoking, use smokeless tobacco, consumption of alcoholic beverages, physical activity level, and mMDS. Logistic regression models were also utilized to estimate the odds ratios (OR) and 95% confidence intervals (CI) for having hypertension and for being obese according to the dichotomous sleep quality and quantity measures. In addition, we performed additional analysis for firefighters reporting short sleep at the firehouse, to compare firefighters who report short sleep at the firehouse and home with firefighters who report short sleep at the firehouse but usual sleep at home (recovery sleep).

The statistical analysis was carried out using SPSS version 24.0 for Windows (SPSS Inc, Chicago, IL) and *p* < 0.05 (two-tailed) was considered as significant.

## Results

A total of 258 participants met all study inclusion criteria (**Supplementary Figure 1**). The firefighters’ scores were ranging from 2 to 17 points. Based on the SQI, the participants were characterized as bad sleepers (SQI>8) and as good sleepers (SQI ≤ 8). One-third of them (31%) reported short sleep duration at home (≤6 hours per night), at firehouse, most of them (79.1%) reported short sleep duration per night, while half of them (54.3%) reported short sleep duration in a 24-hours period including naps. The demographic characteristics of the participants are summarized in **Table 1**, overall and stratified according to sleep quality. The mean age of the participants was 48.8 ± 7.6 years, without significant differences between groups, and the majority were males (94%). The two groups were also similar with respect to socio-demographic factors, adherence to healthy eating habits (assessed by mMDS), physical activity, tobacco smoking and alcohol intake (all p-values >0.05).

**Table 1:** Demographic characteristics of the study population.

	Overall	Good Sleepers (SQI ≤ 8)	Bad Sleepers (SQI>8)	*p*
**N**	258	129 (50.0%)	129 (50.0%)	
**Age**	48.8 ± 7.6	48.1 ± 7.6	49.6 ± 7.6	0.10
**Gender % (Male)**	243 (94.2%)	122 (94.6%)	121 (93.8%)	0.79
**Race %**				
**-** **Caucasian**	215 (83.4%)	105 (81.4%)	110 (85.3%)	0.59
**- African American**	37 (14.3%)	20 (15.5%)	17 (13.2%)	
**- Asian, Native Hawaiian or American Native**	6 (2.3%)	4 (3.1%)	2 (1.5%)	
Highest level of education completed* %				
**- Basic or intermediate education**	130 (50.4%)	58 (45.0%)	72 (55.8%)	0.08
**- Advanced education**	128 (49.6%)	71 (55.0%)	57 (44.2%)	
**Current Marital Status**				
**- Married**	207 (80.3%)	102 (79.1%)	105 (81.4%)	0.84
**- Divorced or widowed**	37 (14.3%)	19 (14.7%)	18 (14.0%)	
**- Never Married**	14 (5.4%)	8 (6.2%)	6 (4.6%)	
**Second job %**				
**- No**	111 (43.0%)	56 (43.4%)	55 (42.6%)	0.92
**- Yes, 1 other job**	119 (46.1%)	60 (46.5%)	59 (45.8%)	
**- Yes, more than one other job**	28 (10.9%)	13 (10.1%)	15 (11.6%)	
**modified Mediterranean Diet Score (mMDS)**	24.3 ± 6.2	24.8 ± 5.6	23.8 ± 6.7	0.20
**Smoke (active) %**	10 (3.9%)	5 (3.9%)	5 (3.9%)	0.99
**Use smokeless tobacco**				
**- Not at all**	234 (91.1%)	119 (92.2%)	115 (89.8%)	0.07
**- Not every day**	8 (3.1%)	6 (4.7%)	2 (1.6%)	
**- Every day**	15 (8.6%)	4 (3.1%)	11 (8.6%)	
**Alcohol beverages per week (beer, wine or spirits)**	3 (3-5)	4 (2-5)	3 (3-5)	0.32
**Physical Activity**				
**- Not practice regularly recreation, sport, or vigorous physical activity**	38 (14.7%)	16 (12.4%)	22 (17.1%)	0.16
**- Practice regularly recreation or work requiring modest physical activity**	61 (23.6%)	26 (20.2%)	35 (27.1%)	
**- Regular vigorous physical exercise**	159 (61.6%)	87 (67.4%)	72 (55.8%)	

*Basic or intermediate education included high school or equivalent, vocational/technical school and some college, and advanced education included associate degree, bachelor’s degree, master’s degree and doctoral degree.

Bad sleepers on average had higher body weight, waist circumference, BMI, as well as body fat (all p<0.01) than good sleepers, but did not have significantly higher systolic and diastolic blood pressure (**Table 2**). In addition, bad sleepers had a higher prevalence of obesity *(p*<0.001), abdominal obesity *(p*<0.001) and hypertension *(p*<0.01), compared with good sleepers (**Table 2**).

**Table 2:** Anthropometrics and hypertension in “good” and “bad” sleepers as classified using the SQI.

	Good Sleepers (n=129/50%)	Bad Sleepers (n=129/50%)	*p*
**Weight Baseline (kg)**	92.8 +/- 14.2	99.0 +/- 17.8	<0.01
**Waist Circumference (cm)**	96.4 +/- 10.5	101.8 +/- 12.7	<0.01
**BMI (kg/m^2^)**	28.8 +/- 3.7	30.8 +/- 4.6	<0.01
**Body Fat %**	26.6 +/- 6.3	29.7 +/- 7.1	<0.01
**Systolic blood pressure**	122.6 +/- 8.6	123.5 +/- 8.3	0.59
**Diastolic blood pressure**	78.1 +/- 6	79.6 +/- 5.5	0.08
**Obesity (≥30 kg/m^2^)**	43 (33.3%)	70 (54.3%)	<0.001
**Abdominal Obesity (≥102 cm for males and ≥88 cm for females)**	62 (27.9%)	36 (48.1%)	<0.001
**HTN %**	61 (47.3%)	83 (64.3%)	<0.01

BMI, body mass index; HTN, hypertension; SQI, Sleep Quality Index.

We also found significant differences between firefighters reporting short sleep and those with normal sleep duration at home (**Table 3**). Short sleepers had higher body fat, BMI, and diastolic blood pressure (all *p*<0.05). Short sleepers also had a higher prevalence of hypertension (*p* 0.02) and obesity (*p* 0.03). On the other hand, firefighters reporting short sleep at firehouses had differences in anthropometric measures and blood pressures compared to those with normal sleep duration, but these differences were not significant (data not shown). Finally, firefighters reporting short sleep in a 24-hours period including naps, had higher weight, BMI, body fat and diastolic blood pressure (*p*<0.05) and a higher prevalence of hypertension (*p*<0.01) (**Supplementary Table 2**). In addition, for firefighters reporting short sleep at the firehouse (n=204) we performed additional analysis to compare whose firefighters reporting short sleep at the firehouse and home (n=77, 37.7%) with firefighters reporting short sleep at the firehouse but usual sleep at home (n=127, 62.3%) recovery sleep. Firefighters reporting recovery sleep had lower body fat (*p*<0.01), BMI (*p* 0.03) and prevalence of hypertension (*p* 0.046) and obesity (*p* 0.016) (**Table 4**).

**Table 3:** Anthropometric measures, obesity and hypertension rates according to nightly home sleep duration (less than 6 hours *versus* more than 6 hours).

	> 6 hours sleep per night (n=178/69%)	≤ 6 hours sleep per night (n=80/31%)	*P*
**Weight Baseline (kg)**	94.6 +/- 16.0	99.0 +/- 16.9	0.13
**Waist Circumference (cm)**	98.0 +/- 11.3	101.5 +/- 13.1	0.09
**BMI**	29.3 +/- 4.1	30.8 +/- 4.6	0.02
**Body Fat %**	27.4 +/- 6.6	29.9 +/- 7.2	<0.01
**Systolic blood pressure**	122.9 +/- 8.8	123.5 +/- 7.7	0.84
**Diastolic blood pressure**	78.2 +/- 6.0	80.2 +/- 5.2	0.03
**Obesity (≥30 kg/m^2^)**	70 (39.3%)	43 (53.8%)	0.03
**Abdominal Obesity (≥102 cm for males and ≥88 cm for females)**	64 (36%)	34 (42.5%)	0.32
**HTN (%)**	91 (51.1%)	53 (66.3%)	0.02

BMI, body mass index; HTN, hypertension.

**Table 4:** Anthropometric measures, obesity and hypertension rates according to reports of post-shift recovery sleep at home.

	Recovery sleep(n=127/62.3%)	≤ 6 hours at firehouse and home (n=77/37.7%)	*P*
**Weight Baseline (kg)**	94.7 +/- 16.3	99.4 +/- 16.9	0.15
**Waist Circumference (cm)**	97.6 +/- 11.4	101.8 +/- 13.1	0.09
**BMI**	29.3 +/- 4.1	31 +/- 4.6	0.03
**Body Fat %**	27.2 +/- 6.6	30.5 +/- 7.3	<0.01
**Systolic blood pressure**	122.7 +/- 9.2	123.5 +/- 7.8	0.79
**Diastolic blood pressure**	78.4 +/- 6.1	80.2 +/- 5.2	0.12
**Obesity (≥30 kg/m^2^)**	49 (38.6%)	43 (55.8%)	0.02
**Abdominal Obesity (≥102 cm for males and ≥88 cm for females)**	44 (34.6%)	33 (42.9%)	0.24
**HTN (%)**	66 (56.4%)	51 (66.2%)	0.04

BMI, body mass index; HTN, hypertension.

We further examined the effect of a unitary increase in the SQI (decreasing sleep quality) on anthropometric variables (weight, waist circumference, body fat % and BMI). We found that a unit increase in SQI was associated with significant increases in weight (β=0.78, *p*=0.01), waist circumference (β=0.58, *p*<0.01), body fat (β=0.42, *p*<0.01), and BMI (β=0.27, *p*<0.01) after adjusting for age, gender, physical activity, smoking, mMDS, and alcohol intake (**Table 5**).

**Table 5:** Effect of a unitary increase in the sleep quality index (SQI) on anthropometric variables.

	Linear regression models								
	Unadjusted			Adjusted for age and gender			Adjusted for age, gender, physical activity, smoking, use smokeless tobacco, modified Mediterranean Diet Score, and alcohol intake		
	B	SE	*P*	β	SE	*p*	β	SE	*P*
**Weight (kg)**	0.95	0.33	<0.01	0.95	0.32	<0.01	0.78	0.32	0.01
**Waist circumference (cm)**	0.82	0.24	<0.01	0.74	0.22	<0.01	0.58	0.21	<0.01
**Body Fat %**	0.58	0.14	<0.01	0.52	0.13	<0.01	0.42	0.12	<0.01
**BMI kg/m^2^**	0.33	0.09	<0.01	0.32	0.09	<0.01	0.27	0.08	<0.01

BMI, body mass index.

In addition, we found that being a bad sleeper was associated with hypertension (OR=1.83, 95% CI: 1.05-3.20), obesity (OR=2.17, 95% CI: 1.23-3.82) and abdominal obesity (OR= 2.15, 95% CI: 1.14-4.05) as compared to good sleepers after adjusting for age, gender, physical activity, mMDS, smoking, and alcohol intake. Similar results were found for participants who slept ≤6 hours per night at home for having hypertension (OR=1.88, 95% CI: 1.02-3.46) or abdominal obesity (OR=1.67, 95% CI: 0.92-3.05) and for being obese (OR=1.14, 95% CI: 0.58-2.21) after adjustment for the same covariates as compared to firefighters who slept >6 hours per night.

## Discussion

Our results suggest that both the quality and quantity of sleep were associated with cardiometabolic risk among Indiana firefighters in the Feeding America’s Bravest cohort. Specifically, we found that bad sleepers had higher weight, waist circumference, body fat and BMI compared with the good sleepers and additionally had a higher prevalence of hypertension and obesity. In addition, those participants who slept 6 or less hours per night at home had higher weight, BMI and hypertension prevalence compared with those who slept more than 6 hours per night. This study provides additional evidence supporting that the quality and quantity of sleep are likely important modifiable cardiometabolic risk factors among firefighters.

Our study is consistent with and expands previous results in firefighters and other shift workers. Shift work has been previously linked with poor quality of sleep and short sleep duration (23, 24). Barger et al. found an association in firefighters between likely sleep disorders such as obstructive sleep apnea (OSA) and insomnia and health outcomes including cardiovascular disease, diabetes, depression or anxiety (7). Similarly, Rajaratnam et al. demonstrated analogous associations in a large cohort of police officers (13). In addition, Liu Q et al. found that shift workers were found to have a higher prevalence of overweight and obesity in a meta-analysis including 311,344 participants (25). Similarly, Chang JH et al. found an association between sleep duration and sleep quality as measured by the Pittsburgh Sleep Quality Index questionnaire and Metabolic Syndrome (MS) in a police officer cohort (26). Therefore, evidence suggests that participants with sleep disturbances may be predisposed to a greater risk of cardiovascular events (27, 28).

Globally, insufficient sleep is considered to be a public health epidemic with serious health implications (29). Nocturnal exposure to light among shift workers along with irregular meal timing has been demonstrated to be important factors driving circadian disruption (30).

The relationship between circadian disruption and obesity is well established (31). The circadian system is responsible for synchronizing energy homeostasis with the day-night cycle as other biological processes are critical for the control of body weight and for general metabolic health (32). In our study, we demonstrated that firefighters with sleep disturbance (quality or quantity) had higher BMI, waist circumference and obesity. Many studies support this relation noting the importance of the short sleep duration stimulates hunger and appetite probably through hormonal mechanisms (33).

With respect to hypertension, we showed that it is associated with poorer quality sleep and insufficient sleep (defined as those who slept less than 6 hours). Over the diurnal cycle, blood pressure is regulated by the circadian system and disruption of this system has been linked with a higher prevalence of hypertension (34).

Additionally, poor sleep status and sleep disorders are linked with lower productivity at work and a greater risk of accidents (29). Therefore, sleep improvement programs providing sleep health education and sleep disorders screenings can likely reduce injuries and disability among firefighters (35).

Barger et al. found that among almost 7,000 US firefighters undergoing sleep disorders screening, the risk of several adverse health and safety outcomes was elevated in those firefighters screening positive for a sleep disorder (7). Nonetheless, future longitudinal studies are needed to demonstrate causation, or if sleep health interventions can reduce future CVD events.

Limitations of this study included the self-reported information from a lifestyle questionnaire that included sleep quality. In addition, the cross-sectional study design limits our ability to establish causal relationships, however there are plausible biologic mechanisms that support the associations found. In addition, OSA is the most common sleep disorder among firefighters and is associated with obesity and hypertension. This could explain some of the associations we found but is also consistent with sleep disruption being associated with increased CVD risk. Our sleep quality index has not been validated. Additionally, the generalizability of our findings could be limited because we studied firefighters from a single state. The main strength of the current work was the independent objective assessments of the outcome measures.

Our results showed that poor sleep quality and sleep quantity were both associated with more adverse cardiometabolic risk profiles among career firefighters. This study contributes to the growing evidence that sleep quality and quantity are modifiable cardiovascular risk factors and encourages improved sleep hygiene to reduce CV burden in this shift working population.

## Data Availability Statement

The datasets used and/or analysed during the current study are available from the corresponding author on reasonable request.

## Ethics Statement

The studies involving human participants were reviewed and approved by Harvard Institutional Review Board (IRB16-0170). The patients/participants provided their written informed consent to participate in this study.

## Author Contributions

JLRC and AGR contributed to the design of the study, analysis and interpretation of data, and drafting the manuscript. MSP and PPM contributed to study design, acquisition of data, interpretation of data, and drafting the manuscript. SM contributed to obtaining funding and acquisition of data. CAC contributed to the analysis and interpretation of data, and SNK conceived the idea and the design of the study, contributed to obtaining funding, acquisition of data, analysis and interpretation of data, and drafting the manuscript. All authors contributed to the article and approved the submitted version.

## Funding

This study was funded by US Department of Homeland Security [grant number EMW-2014-FP- 0612]. JLRC received grants from the Spanish Internal Medicine Society (SEMI) and the regional government of Andalucía (Spain).

## Conflict of Interest

The authors declare that the research was conducted in the absence of any commercial or financial relationships that could be construed as a potential conflict of interest.

## Publisher’s Note

All claims expressed in this article are solely those of the authors and do not necessarily represent those of their affiliated organizations, or those of the publisher, the editors and the reviewers. Any product that may be evaluated in this article, or claim that may be made by its manufacturer, is not guaranteed or endorsed by the publisher.
