# Supplementary figures and images for: Sleep and Association With Cardiovascular Risk Among Midwestern US Firefighters
Source: Front Endocrinol (Lausanne). 2021 Nov 11;12:772848. doi: 10.3389/fendo.2021.772848 (PMC8632221; doi:10.3389/fendo.2021.772848)

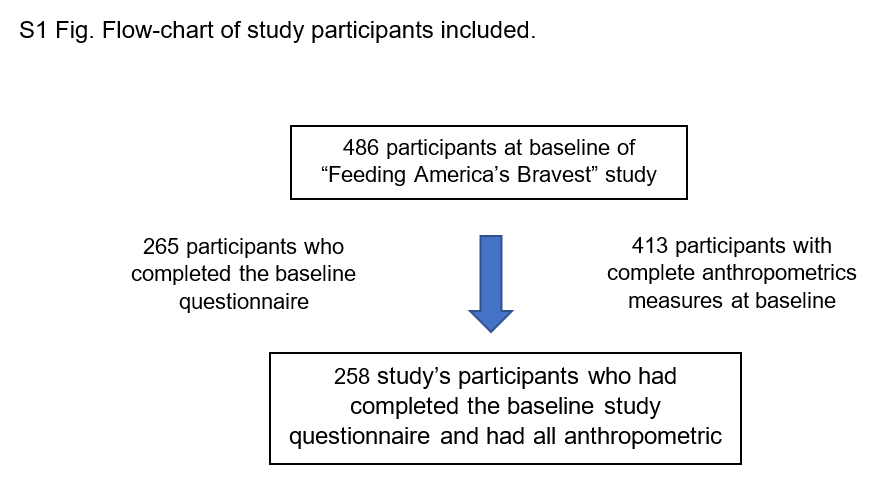

Supplement: Supplementary file 1 [file Image_1.tif]
