# Supplementary material for: Sleep and Association With Cardiovascular Risk Among Midwestern US Firefighters
Source: Front Endocrinol (Lausanne). 2021 Nov 11;12:772848. doi: 10.3389/fendo.2021.772848 (PMC8632221; doi:10.3389/fendo.2021.772848)
Supplement: Supplementary file 2 [file Table_1.docx]

Table 1 Supplementary Data Sleep Behaviors: Assessed by the Sleep Quality Index (SQI).

| In the following set of questions, please select the answer choices that most closely describe your sleep behaviours. | 1. Please indicate the total hours of actual sleep in a typical 24- hour period: 5 or less (2 points), 6 hours (1 points), 7 hours or 8 hours (0 points), 9 hours (1 points), 11 + hours (2 points) |
| --- | --- |
|  | 1. How many hours do you sleep most nights at home? <4 hours or 4-5 hours (2 points), 5-6 hours (1 points), 6-7 hours or 7-8 hours (0 points), 8-9 hours (1 points), > 9 hours (2 points) |
|  | 1. How many hours do you sleep most nights at the firehouse? <4 hours or 4-5 hours (2 points), 5-6 hours (1 points), 6-7 hours or 7-8 hours (0 points), 8-9 hours (1 points), > 9 hours (2 points) |
|  | 1. During the past month, how many nights per week have you used medications to help you sleep? Never or 1 night (0 points), 2-4 nights (1 point), 5-6 nights (2 point), Every night (3 points) |
|  | 1. How many times do you take a nap per week? Never or 1 or less times (3 points), 2-3 times (2 points), 3-4 times (1 points), 5-6 times or every day (0 points) |
| Please indicate how often you experience or do the following (frequency: rarely or never 0 points, sometimes 1 point, most of the time 2 points) | 1. Trouble falling asleep |
|  | 1. Trouble staying asleep |
|  | 1. Waking up too early and not being able to fall back asleep |
|  | 1. So sleepy during the day or evening that you have to take a nap |
|  | 1. Feel really rested when you wake up in the morning |
|  | 1. Snore |
| Good Sleepers ≤8 points  Bad Sleepers >8 points | |
