# Supplementary material for: Sleep and Association With Cardiovascular Risk Among Midwestern US Firefighters
Source: Front Endocrinol (Lausanne). 2021 Nov 11;12:772848. doi: 10.3389/fendo.2021.772848 (PMC8632221; doi:10.3389/fendo.2021.772848)
Supplement: Supplementary file 3 [file Table_2.docx]

Supplementary Table 2 Anthropometrics and Hypertension, firefighters who sleep less than 6 hours in 24-hours period including naps.

|  | > 6 hours  (n=118/45.7%) | ≤ 6 hours (n=140/54.3%) | *P* |
| --- | --- | --- | --- |
| Weight Baseline (kg) | 93.3 +/- 14.9 | 98.1 +/- 17.2 | 0.04 |
| Waist Circumference (cm) | 97.6 +/- 11.1 | 100.3 +/- 12.5 | 0.05 |
| BMI | 29.1 +/- 3.9 | 30.4 +/- 4.6 | 0.03 |
| Body Fat % | 27.2 +/- 6.4 | 29.0 +/- 7.1 | 0.05 |
| Systolic blood pressure | 122.2 +/- 9 | 123.9 +/- 8 | 0.11 |
| Diastolic blood pressure | 77.8 +/- 6.1 | 79.7 +/- 5.4 | <0.01 |
| Obesity (≥30 kg/m^2^) | 45 (38.1%) | 68 (48.6%) | 0.09 |
| Abdominal Obesity (≥102 cm for males and ≥88 cm for females) | 42 (35.6%) | 56 (40%) | 0.47 |
| HTN (%) | 54 (45.8%) | 90 (64.3%) | <0.01 |

**Abbreviations:** BMI, body mass index; HTN, hypertension
